# Reduction of radiation dose to the eye lens during common CT examinations of the head, paranasal sinus, and cervical spine in emergency settings: A phantom study

**DOI:** 10.1002/acm2.70486

**Published:** 2026-02-09

**Authors:** Sowitchaya Huakham, Wirachad Sripoori, Raksumon Suksi, Thawatchai Thaikan, Thanyawee Pengpan

**Affiliations:** ^1^ Department of Radiological Technology Faculty of Allied Health Sciences Thammasat University Pathumthani Thailand; ^2^ Department of Radiology King Chulalongkorn Memorial Hospital Thai Red Cross Society Bangkok Thailand; ^3^ Department of Radiological Technology Faculty of Allied Health Sciences Naresuan University Mueang Phitsanulok Thailand

**Keywords:** automatic tube current modulation, computed tomography, eye lens dose, head and neck CT, image quality, organ dose modulation, radiation dose

## Abstract

**Background:**

Computed tomography (CT) examinations of the head, paranasal sinus (PNS), and cervical spine (C‐spine) are frequently performed in emergency settings, raising concerns about radiation exposure to the radiosensitive eye lens. Overexposure can cause radiation‐induced ocular damage. To address this concern, organ dose modulation (ODM) has emerged as a promising technique for reducing eye lens dose in CT examinations.

**Purpose:**

This study aimed to evaluate radiation exposure to the eye lens and objective image quality metrics for head, PNS, and C‐spine CT examinations using fixed tube current, automatic tube current modulation (ATCM), and ODM techniques.

**Methods:**

Eye lens doses were measured using nanoDot optically stimulated luminescence dosimeters (OSLDs) placed bilaterally to the eye lens of a whole‐body anthropomorphic phantom (PBU‐60). CT scans were performed using a Revolution EX CT scanner with three scanning techniques: fixed tube current, ATCM, and ODM. The phantom was scanned twice for each examination type (head, PNS, and C‐spine) with all three techniques. Eye lens dose reductions with the ODM technique were quantified relative to fixed tube current and ATCM techniques. Image quality was quantitatively evaluated in terms of image noise, signal‐to‐noise ratio (SNR), and contrast‐to‐noise ratio (CNR).

**Results:**

Mean eye lens doses ± standard deviation (SD) using the ODM technique were 38.44 ± 1.37, 17.92 ± 1.01, and 9.77 ± 0.38 mGy for head, PNS, and C‐spine, respectively. These eye lens doses were reduced by 4.28%, 21.33%, and 47.97% compared to the fixed tube current techniques and by 19.40%, 24.70%, and 13.69% compared to the ATCM techniques, for head, PNS, and C‐spine, respectively. These dose reductions were achieved while maintaining image quality metrics (image noise, SNR, and CNR) with no statistically significant differences (*p* > 0.05) compared to fixed tube current and ATCM techniques.

**Conclusion:**

Implementation of the ODM technique resulted in significant eye lens dose reduction (4.28%–47.97%) across head, PNS, and C‐spine CT examinations with no significant differences in image noise, SNR, and CNR compared to both fixed tube current and ATCM techniques. ODM demonstrates potential as a practical dose optimization strategy for routine emergency head and neck CT imaging. Further studies with subjective image quality assessment are recommended to evaluate clinical diagnostic acceptability in hospital settings.

## 1 INTRODUCTION

Computed tomography (CT) has become an essential diagnostic tool in modern medical practice, particularly in emergency settings where rapid detection of internal injuries and hemorrhage is critical for timely patient management. The utilization of CT has increased substantially over recent decades. The National Council on Radiation Protection and Measurements (NCRP) reported a 20% increase in CT examinations in the United States between 2006 and 2016.^1^ CT contributes to about 63% of the collective effective dose from non‐therapeutic medical radiation dose, which increased from 50% in 2006.^1^, ^2^, ^3^

Head CT is the most frequently requested CT examination in emergency settings.^4^, ^5^ Paranasal sinus (PNS) CT is commonly performed for suspected sinusitis and facial trauma, while cervical spine (C‐spine) CT has become the dominant imaging modality for C‐spine evaluation following dramatic increases in utilization.^6^ From a radiation protection perspective, these examinations are of particular concern because the eye lens—a critical radiosensitive organ—lies directly within or adjacent to the primary x‐ray beam.

The eye lens is located on the anterior aspect of the facial bones in the head and neck region. Previous studies indicate that entrance surface doses to the eye lens during head CT examinations can range from 33.62 to 51.80 mGy.^7^, ^8^ Radiation‐induced cataract formation is a primary concern for patients undergoing head and neck CT examinations.^9^ In 2011, the International Commission on Radiological Protection (ICRP) established a threshold for absorbed dose of 0.5 Gy for radiation exposure to the lens of the eye.^10^

To address this concern, several strategies have been proposed to reduce eye lens exposure. While physical gantry tilting is one of the most effective methods, with Nikupaavo et al. demonstrating an eye lens dose reduction of over 75%.^11^ However, this technique is limited by specific CT scanner designs.^12^, ^13^ Another approach, the application of bismuth shielding, has also been employed.^14^ This method, however, could degrade image quality due to streak artifacts from the high atomic number of bismuth and potential interference with critical anatomical structures from improper shield placement.^15^, ^16^, ^17^ Given these limitations, organ dose modulation (ODM) technique was recently developed as an effective alternative method for reducing radiation exposure to radiosensitive organs in the anterior region, including the eyes, breasts, and thyroid.^18^, ^19^, ^20^, ^21^ This technique modulates the tube current based on angular position, effectively reducing the tube current during the anterior 90° arc of the scan,^18^, ^22^ while maintaining standard tube current levels during posterior projections to preserve image quality.

In the emergency department, CT examinations are performed under time constraints that often preclude the implementation of advanced dose optimization strategies. ODM offers considerable promise as a readily implementable dose reduction strategy that requires minimal modifications in clinical workflow. However, its clinical application remains largely confined to routine head imaging protocols. PNS and C‐spine CT examinations represent particularly relevant candidates for ODM implementation, given that the eye lens is located within or immediately adjacent to the primary radiation field in these protocols. Despite this potential, published literature on ODM has predominantly focused on head CT examinations,^23^, ^24^, ^25^ with limited investigation into other head and neck protocols commonly performed in emergency settings.

Furthermore, while previous ODM studies for head CT have demonstrated substantial eye lens dose reductions and acknowledged potential increases in image noise due to reduced anterior tube current,^19^, ^26^, ^27^, ^28^ a comprehensive assessment of image quality metrics, such as signal‐to‐noise ratio (SNR) and contrast‐to‐noise ratio (CNR), has not been extended to other head and neck protocols. Despite these potential dosimetric benefits, two critical knowledge gaps remain: first, the dose reduction achievable with ODM in PNS and C‐spine protocols has not been systematically quantified; second, the impact of reduced anterior tube current on diagnostic image quality remains inadequately investigated. This lack of comprehensive evaluation limits the ability to establish protocol‐specific quality assurance benchmarks and may contribute to reluctance in adopting ODM beyond routine head imaging.

To address these gaps, the present study aims to evaluate eye lens radiation dose and objective image quality (image noise, SNR, CNR) metrics for head, PNS, and C‐spine CT examinations performed using fixed tube current, automatic tube current modulation (ATCM), and ODM techniques. A CT whole‐body anthropomorphic phantom (PBU‐60) was employed to ensure standardized and reproducible measurements across protocols.

## 2 MATERIALS AND METHODS

### 2.1 CT examination

**FIGURE 1:**
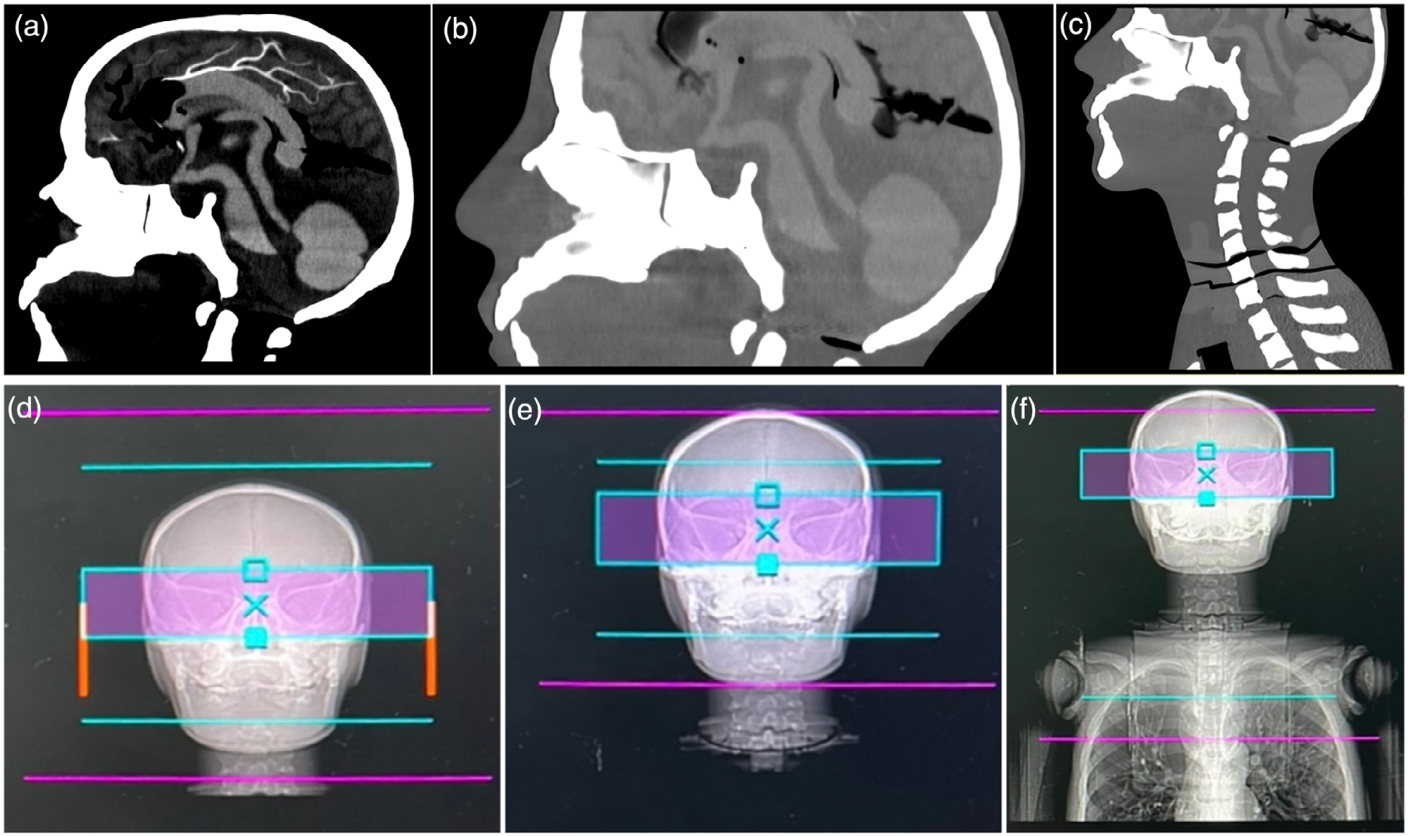
CT scan ranges and ODM activation zones for head, PNS, and C‐spine examinations. (a) Head CT scan ranges from the vertex to the skull base. (b) PNS CT scan ranges from above the frontal sinuses to the hard palate. (c) C‐spine CT scan ranges from the skull base to the first thoracic vertebra. (d–f) Activated ODM zones (pink bands) are positioned to cover the orbital level in head, PNS, and C‐spine CT examinations, respectively.

A CT whole‐body anthropomorphic phantom (PBU‐60; Kyoto Kagaku Co., Ltd., Kyoto, Japan) was scanned using a Revolution EX CT scanner (GE Healthcare; Milwaukee, WI, USA). Three clinical protocols were evaluated: head, PNS, and C‐spine CT examinations. Complete technical specifications, including tube voltage (kV), rotation time, pitch factor, detector configuration, and scan length, are provided in Table 1. Three scanning techniques were employed for each CT examination: (1) fixed tube current, (2) ATCM, and (3) ODM. To ensure measurement reproducibility and reliability, the phantom was scanned twice for each examination type (head, PNS, and C‐spine) using all three scanning techniques, resulting in a total of 18 acquisitions. For ODM implementation, the orbital bone served as the anatomical landmark for the eye lens. The ODM activation zone was defined as a 5‐cm region centered on the orbital bone, providing adequate coverage while minimizing interference with adjacent anatomical structures (Figure 1). The CT images obtained from all three scanning techniques for head, PNS, and C‐spine CT examinations are presented in Figure S1.

**TABLE 1:** CT acquisition parameters for head, PNS, and C‐spine using fixed tube current, ATCM, and ODM.

Parameters	CT examination								
	Head			PNS			C‐spine		
	Fixed tube current	ATCM	ODM	Fixed tube current	ATCM	ODM	Fixed tube current	ATCM	ODM
Acquisition mode	Helical			Helical			Helical		
Tube voltage (kV)	120			120			120		
Tube current (mA)	320	220–440		180	80–400		180	120–400	
Collimation (mm)	40.00 (64 × 0.625)			40.00 (64 × 0.625)			40.00 (64 × 0.625)		
Rotation time (s)	0.50			1.00			0.50		
Field of view (mm^2^)	Head (302 × 240)			Head (302 × 240)			Head (302 × 240)		
Pitch	0.516			0.984			0.516		
Noise index	2.50			3.50			3.50		
ODM	Inactive	Inactive	Active	Inactive	Inactive	Active	Inactive	Inactive	Active
Slice thickness (mm)	5.00			5.00			5.00		
Number of slices	38.00			25.00			51.00		
Scan range (mm)	190.00 (I^*^65‐S^**^125)			125.00 (I^*^50‐S^**^75)			255.00 (I^*^75‐S^**^180)		
Scan range	The vertex to the base of the skull			Above the superior aspect of the frontal sinuses to the hard palate			The base of the skull to the first thoracic vertebra		
Image reconstruction	DLIR‐M^***^			DLIR‐M^***^			DLIR‐M^***^		

* Inferior border (I).

** Superior border (S).

*** Deep learning image reconstruction‐Medium (DLIR‐M).

### 2.2 Radiation dose measurement

**FIGURE 2:**
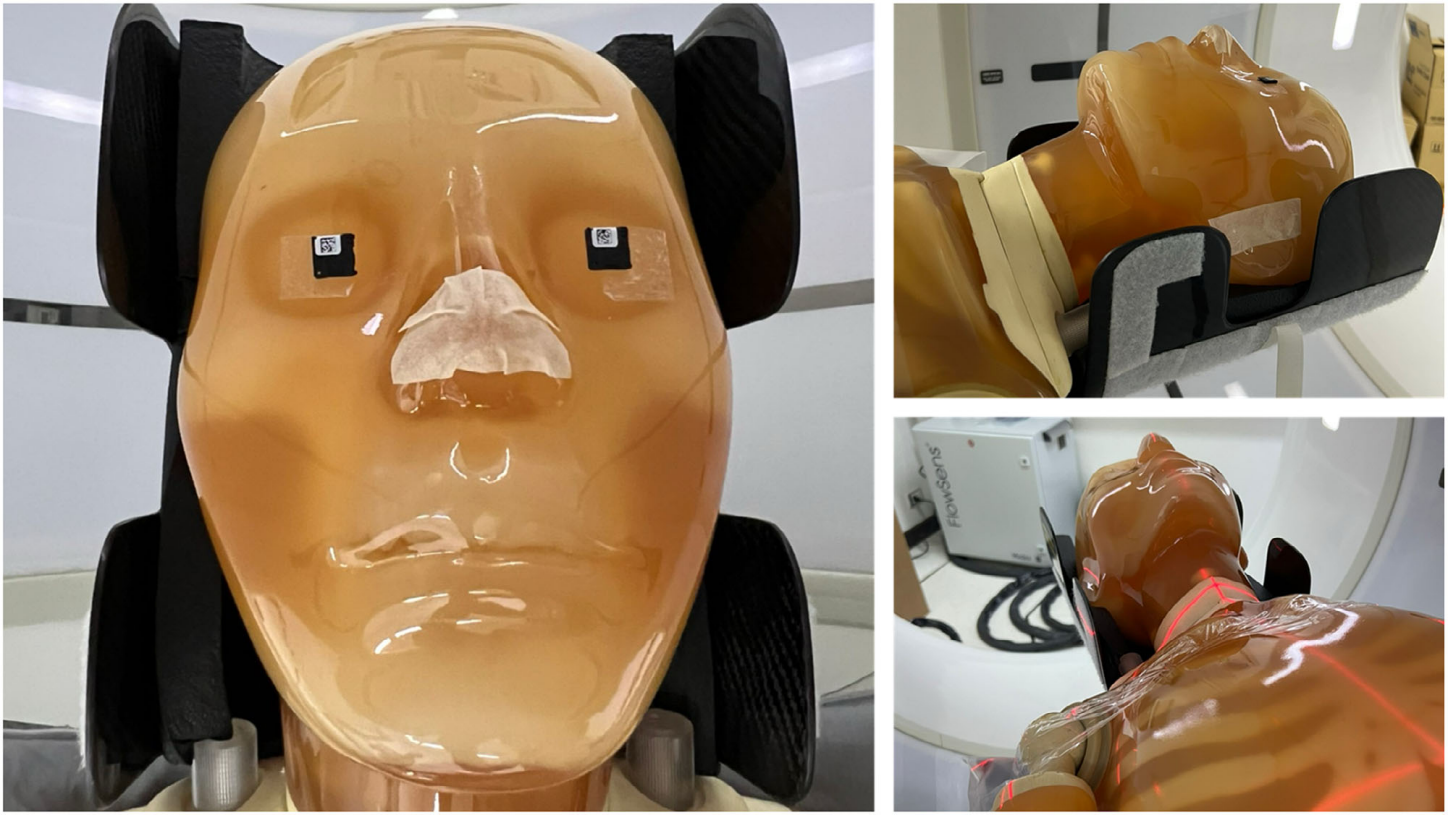
Experimental setup for eye lens dose measurements. NanoDot OSLDs were placed on both eyes of the whole‐body anthropomorphic phantom (PBU‐60) for head, PNS, and C‐spine CT examinations using fixed tube current, ATCM, and ODM techniques.

Eye lens dose was measured using optically stimulated luminescence dosimeters (OSLDs) (NanoDot™, Landauer, Inc., Illinois, USA). The nanoDot™ dosimeter is made from carbon‐doped aluminum oxide (Al_2_O_3_:C) embedded in a plastic matrix. The OSLD crystals are 5 mm in diameter and 0.3 mm in thickness. OSLDs were encased in plastic envelopes (density = 1 g/cm^3^) with dimensions of 10 × 10 × 2 mm^3^.^29^ OSLDs were placed on the left and right eyes of the phantom for eye lens dose measurement, as illustrated in Figure 2.

Dose measurements were performed independently for all three scanning techniques. After irradiation, all OSLDs were stored at room temperature for approximately 12 h to ensure complete electron‐trapping saturation. Each OSLD was read using a microSTAR ii automated reader system (Landauer, Inc., Glenwood, IL, USA), with five consecutive measurements performed, yielding 20 total measurements (two OSLDs, two scans, five readings) for each scanning condition. These multiple readouts quantified measurement uncertainty and improved statistical precision. The average doses were calculated using Equation (1). In addition to OSLD measurements, standard CT dose indices, including volume CT dose index (CTDI_vol_) and dose‐length product (DLP), were recorded from scanner‐generated dose reports. The values were averaged from two repeated scans for each scanning protocol to ensure measurement reliability.

(1) $$\mathrm{Eye}\;\mathrm{lens}\;\mathrm{dose}\;\left(\mathrm{mGy}\right)=\frac{\mathrm{average}\;\mathrm{raw}\;\mathrm{counts}\;-\;\mathrm{corrected}\;\mathrm{background}\;\mathrm{counts}}{\mathrm{reader}\;\mathrm{calibration}\;\mathrm{factor}\;x\;\mathrm{sensitivity}\;x\;\mathrm{sensitivity}\;\mathrm{adjustment}\;\mathrm{factor}}\;$$

The reader calibration factor represents the conversion of raw PMT counts to absorbed radiation dose, while the sensitivity refers to the individual response of each OSLD to a specific radiation dose. The sensitivity adjustment factor is set to a default value of 1.0 for all OSLDs.

To account for the energy dependence of OSLD response, all measured values were corrected using a CT‐specific energy correction factor of 1.12 for 120 kV (Equation 2), in accordance with the manufacturer's calibration protocol for poly‐energetic CT spectra.^30^, ^31^ The final eye lens dose for each scanning condition was determined as the arithmetic mean of the energy‐corrected doses measured bilaterally at the left and right eye positions.

(2) $$\mathrm{Final}\;\mathrm{eye}\;\mathrm{lens}\;\mathrm{dose}\;\left(\mathrm{mGy}\right)=\mathrm{eye}\;\mathrm{lens}\;\mathrm{dose}\;x\;1.12$$

### 2.3 Image quality evaluation

Quantitative image quality evaluation was performed by measuring image noise, SNR, and CNR. All images were analyzed using AW Server 3.2 Ext. 6.0 (GE Healthcare, Chicago, IL, USA).

For head and PNS images, five circular regions of interest (ROIs) of 200 mm^2^ were positioned on gray and white matter (object ROIs)^26^ for three different axial slices within the activated ODM zone, resulting in a total of 15 object ROIs for each scanning condition. Two additional circular ROIs of 200 mm^2^ were placed over both eyeballs as background ROIs.

For the C‐spine examinations, image quality was assessed at three different axial slices. One slice was selected within the activated ODM zone; the other two slices were placed outside this zone to assess image quality in the diagnostic region of the C‐spine. Five circular ROIs of 200 mm^2^ were placed on homogeneous soft tissue areas within each slice as object ROIs. Two additional circular ROIs of 200 mm^2^ were placed over both eyeballs as background ROIs.

ROI placement was performed by a single observer to ensure consistency. All ROIs for each image set were triplicated into each scanning technique at the same slice. Image noise, expressed in Hounsfield units (HU), was defined as the mean standard deviation (SD) of pixel values obtained from all object ROIs. SNR and CNR were calculated by using Equations (3) and (4), respectively.^32^

(3) $$\mathrm{SNR}=\frac{\mathrm{Mean}\;\mathrm{pixel}\;\mathrm{value}\;\mathrm{of}\;\mathrm{object}\;\mathrm{ROIs}}{\mathrm{Standard}\;\mathrm{deviation}\;\mathrm{of}\;\mathrm{pixel}\;\mathrm{value}\;\mathrm{of}\;\mathrm{object}\;\mathrm{ROI}s}$$

(4) $$\mathrm{CNR}=\frac{\left|\mathrm{Mean}\;\mathrm{pixel}\;\mathrm{value}\;\mathrm{of}\;\mathrm{object}\;\mathrm{ROIs}\;-\;\mathrm{Mean}\;\mathrm{pixel}\;\mathrm{value}\;\mathrm{of}\;\mathrm{background}\;\mathrm{ROIs}\right|}{\sqrt{\frac{\mathrm{Standard}\;\mathrm{deviation}\;\mathrm{of}\mathrm{pixel}\;\mathrm{value}\;\mathrm{of}\;\mathrm{object}\;\mathrm{ROI}s^{2}+\mathrm{Standard}\;\mathrm{deviation}\;\mathrm{of}\mathrm{pixel}\;\mathrm{value}\;\mathrm{of}\;\mathrm{background}\;\mathrm{ROI}s^{2}}{2}}}$$

### 2.4 Statistical analysis

All statistical analyses were performed using JASP version 0.19.5 (University of Amsterdam, Amsterdam, The Netherlands). Eye lens dose, CTDI_vol_, and DLP were expressed as mean ± SD, whereas image quality metrics (image noise, SNR, and CNR) were reported as mean ± standard error (SE). Data normality was assessed using the Shapiro‐Wilk test. For normally distributed data, one‐way ANOVA with a Bonferroni post hoc test was applied to evaluate differences among the three scanning techniques. For non‐normally distributed data, the Kruskal–Wallis test with Dunn's post hoc correction was used. Left and right eye lens doses were compared using the Wilcoxon signed‐rank test. A *p*‐value < 0.05 was considered statistically significant.

## 3 RESULTS

### 3.1 Eye lens dose, CTDI_vol_, and DLP

Eye lens dose, CTDI_vol_, and DLP for head, PNS, and C‐spine CT examinations using three different techniques (fixed tube current, ATCM, and ODM) are presented in Table 2.

**TABLE 2:** Mean eye lens dose, CTDI_vol_, and DLP for fixed tube current, ATCM, and ODM.

CT examination	Eye lens dose (mGy) (*n* = 20)			CTDI_vol_ (mGy) (*n* = 2)			DLP (mGy·cm) (*n* = 2)		
	Fixed tube current	ATCM	ODM	Fixed tube current	ATCM	ODM	Fixed tube current	ATCM	ODM
Head	40.19 ± 0.74	47.70 ± 2.24	38.44 ± 1.37	46.90 ± 0.00	49.37 ± 0.00	47.34 ± 0.16	1082.74 ± 0.00	1139.89 ± 0.04	1092.82 ± 3.72
PNS	22.78 ± 0.48	23.79 ± 1.95	17.92 ± 1.01	27.64 ± 0.00	25.47 ± 0.00	24.02 ± 0.28	425.80 ± 0.00	392.47 ± 0.04	370.04 ± 4.25
C‐Spine	18.77 ± 0.53	11.31 ± 0.41	9.77 ± 0.38	21.98 ± 0.00	19.85 ± 0.01	19.57 ± 0.03	650.36 ± 0.00	587.14 ± 0.44	578.91 ± 0.83

**FIGURE 3:**
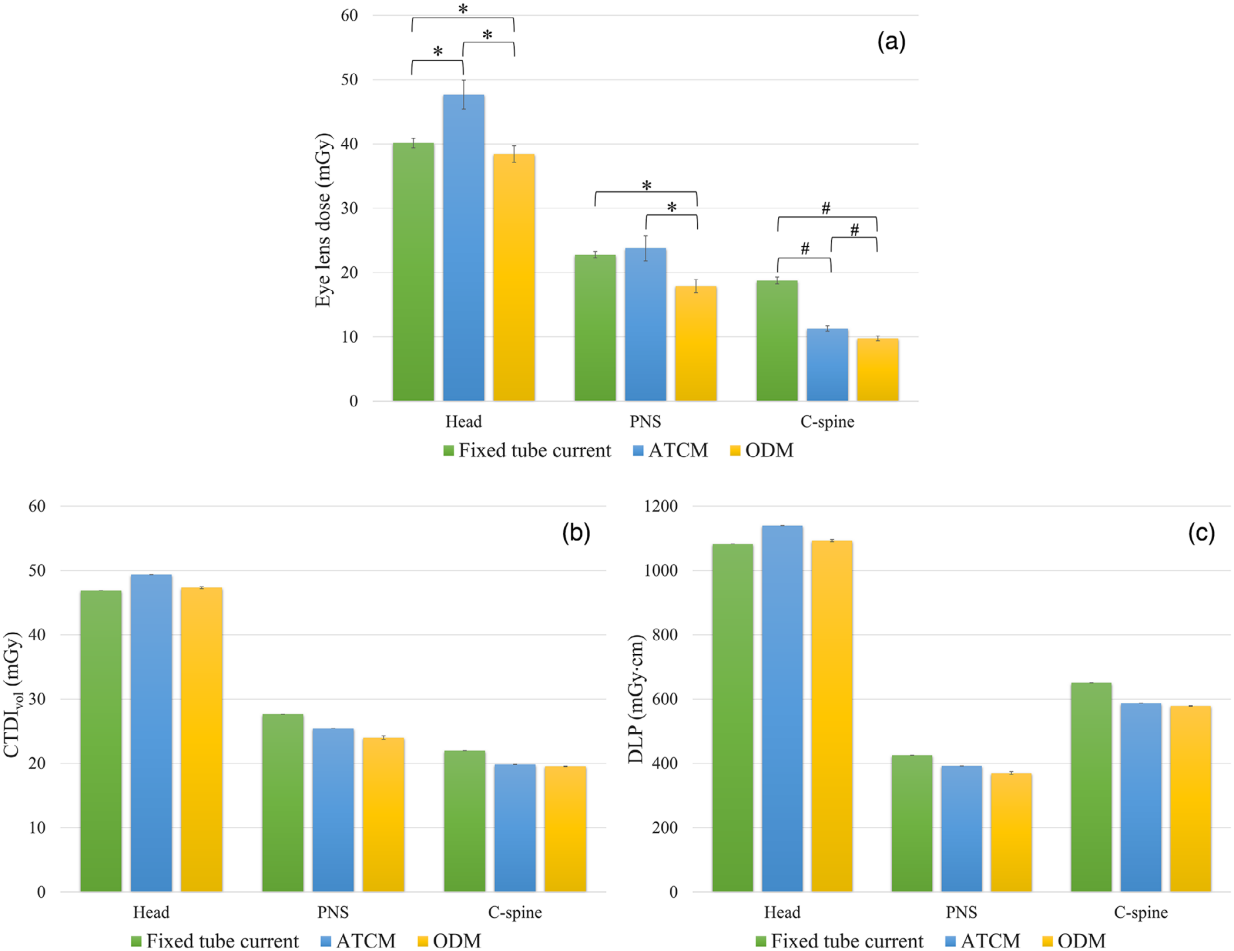
Comparison of radiation dose metrics among three scanning techniques. (a) Eye lens dose, (b) CTDI_vol_, and (c) DLP for head, PNS, and C‐spine CT examinations performed with fixed tube current, ATCM, and ODM techniques. Data are presented as mean ± SD (n = 20 measurements per scanning condition for eye lens dose; *n* = 2 for CTDI_vol_ and DLP). Asterisks (*) indicate statistically significant differences by Kruskal–Wallis test (*p* < 0.05), and hash marks (#) indicate statistically significant differences by one‐way ANOVA (*p* < 0.05). The eye lens dose achieved from the ODM technique was significantly lower than that of the fixed tube current and ATCM techniques.

For head CT, statistically significant differences in eye lens dose were observed between all three techniques: fixed tube current versus ATCM (*p* < 0.001), fixed tube current versus ODM (*p* = 0.011), and ATCM versus ODM (*p* < 0.001) (Figure 3). CTDI_vol_ and DLP demonstrated a decreasing trend from ATCM to ODM to fixed tube current.

In PNS CT examinations, the ODM technique resulted in significantly lower eye lens doses compared to both the fixed tube current technique (*p* < 0.001) and the ATCM technique (*p* < 0.001). CTDI_vol_ and DLP demonstrated an increasing trend across the three techniques, with the lowest values observed for ODM, followed by ATCM, and the highest for fixed tube current.

Similarly, for C‐spine CT, the ODM technique achieved significantly lower eye lens doses than the fixed tube current (*p* < 0.001) and ATCM (*p* < 0.001). CTDI_vol_ and DLP showed a similar trend observed in PNS CT across all three techniques.

Across all three techniques, ODM yielded the lowest eye lens doses, achieving statistically significant reductions compared to both fixed tube current and ATCM techniques (*p* < 0.05). As detailed in Table 3, ODM provided dose reductions ranging from 4.28% to 47.97% across all head, PNS, and C‐spine examinations. The maximum dose reduction was found from the ODM technique compared to the fixed tube current technique for C‐spine CT.

**TABLE 3:** Mean eye lens dose and percentage dose reduction among fixed tube current, ATCM, and ODM.

CT examination	Eye lens dose (mGy)			Fixed tube current versus ODM		ATCM versus ODM	
	Fixed tube current	ATCM	ODM	% Dose reduction	*p* value	% Dose reduction	*p* value
Head	40.19 ± 0.74	47.70 ± 2.24	38.44 ± 1.37	4.28	0.011	19.40	<0.001
PNS	22.78 ± 0.48	23.79 ± 1.95	17.92 ± 1.01	21.33	<0.001	24.70	<0.001
C‐spine	18.77 ± 0.53	11.31 ± 0.41	9.77 ± 0.38	47.97	<0.001	13.69	<0.001

Additionally, there were no statistically significant differences in mean eye lens doses between left and right sides across all CT examinations: head (42.15 ± 4.50 vs. 42.05 ± 4.23 mGy, *p* = 0.853), PNS (21.39 ± 3.45 vs. 21.61 ± 2.24 mGy, *p* = 0.465), and C‐spine (13.25 ± 4.03 vs. 13.31 ± 4.01 mGy, *p* = 0.600), as shown in Table 4. These findings demonstrate bilateral symmetry in dose distribution throughout the phantom.

**TABLE 4:** Comparison of left and right mean eye lens dose in head, PNS, and C‐spine CT.

CT examination	Eye lens dose (mGy)		*p* value
	Left	Right	
Head	42.15 ± 4.50	42.05 ± 4.23	0.853
PNS	21.39 ± 3.45	21.61 ± 2.24	0.465
C‐spine	13.25 ± 4.03	13.31 ± 4.01	0.600

### 3.2 Image quality evaluation

**FIGURE 4:**
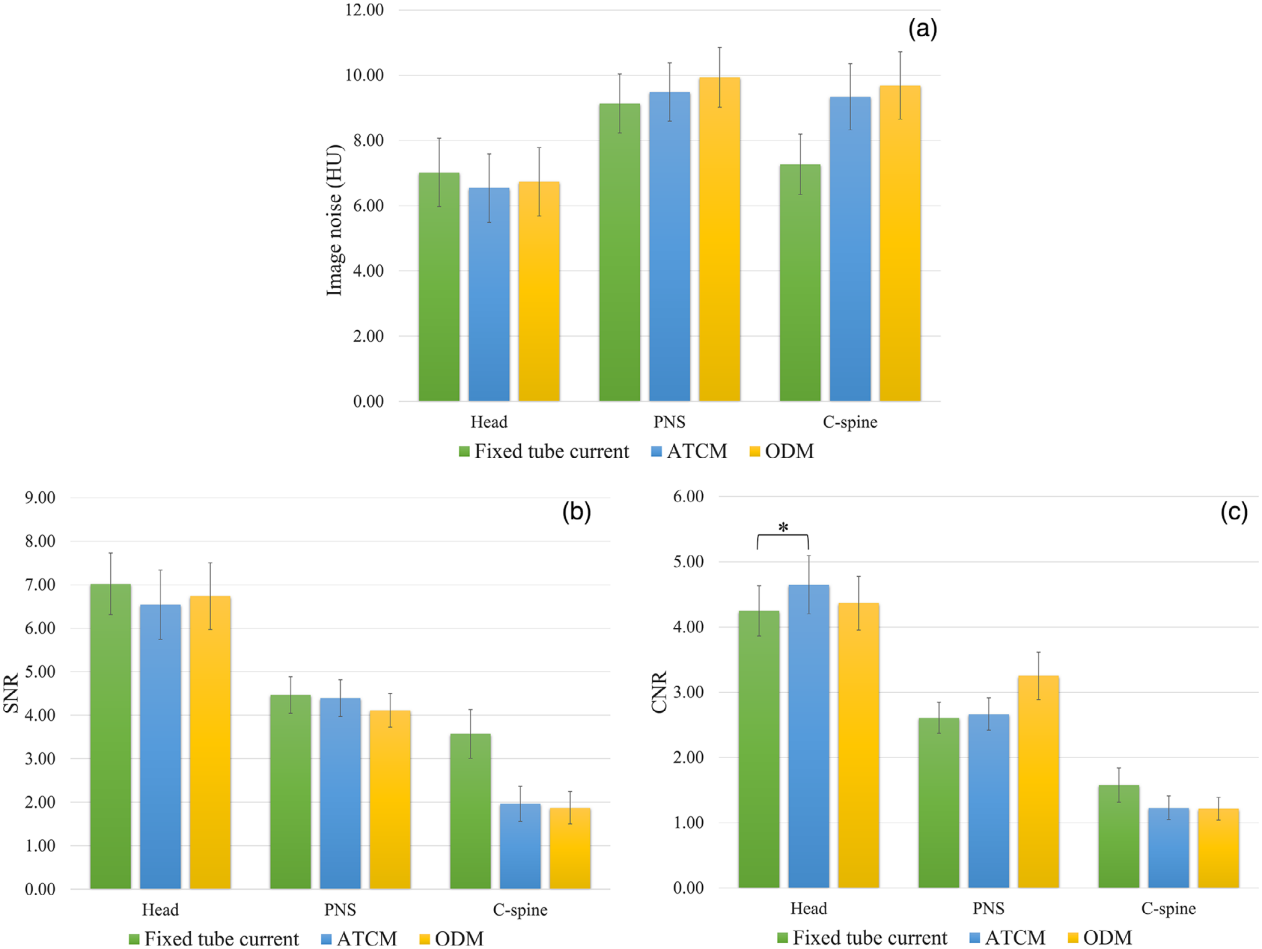
Comparison of image quality metrics among three scanning techniques. (a) Image noise, (b) SNR, and (c) CNR for head, PNS, and C‐spine CT examinations performed with fixed tube current, ATCM, and ODM techniques. Data are presented as mean ± SE (*n* = 15 ROIs per examination). Statistical comparisons were performed using the Kruskal–Wallis test with Dunn's multiple comparison test or one‐way ANOVA with Bonferroni post hoc test (*p* < 0.05). There were no statistically significant differences (*p* > 0.05) in image noise, SNR, and CNR of the ODM technique compared to the fixed tube current and ATCM techniques.

As illustrated in Figure 4, image noise, SNR, and CNR demonstrated no statistically significant differences among the three techniques for all CT examinations (*p* > 0.05), except for CNR in head CT, which was significantly higher with ATCM compared to fixed tube current (*p* = 0.029). These findings indicate that the ODM technique maintains image quality comparable to both the fixed tube current and ATCM techniques. All Image noise, SNR, and CNR data with *p*‐value comparisons among fixed tube current, ATCM, and ODM techniques for head, PNS, and C‐spine CT examinations are presented in Table S1.

## 4 DISCUSSION

The ODM technique was applied to several routine clinical protocols where the eye lens is located within or near the CT scan range. The clinical benefit of lens dose reduction must be balanced against potential compromises in image quality. This study demonstrated that the use of the ODM technique for head, PNS, and C‐spine CT examinations resulted in eye lens dose reductions ranging from 4.28% to 47.97% compared to fixed tube current and ATCM techniques, while maintaining appropriate image quality. The percentage of dose reduction is dependent on the specific body part being examined. The maximum eye lens dose reduction of 47.97% was achieved with the ODM technique compared to the fixed tube current technique for C‐spine CT examination. While the 4.28% reduction in head CT, obtained from ODM compared to fixed tube current, is modest compared to PNS (21.33%) and C‐spine (47.97%), this still represents meaningful dose savings when considering the high frequency of head CT examinations in emergency settings

Although standard CT dose metrics (CTDI_vol_ and DLP) serve as useful indicators of scanner output and overall radiation exposure, they do not accurately quantify organ‐specific absorbed doses. CTDI_vol_ primarily reflects scanner radiation output in a standardized phantom, while DLP represents the product of CTDI_vol_ and scan length. Consequently, direct measurement of eye lens dose remains essential for head and neck CT examinations, particularly given the organ's high radiosensitivity and its superficial location within or adjacent to the primary beam.^33^, ^34^, ^35^ Alwasiah et al. reported a mean eye lens dose of 33.62 ± 12.44 mGy for adult head CT using NanoDot dosimeters,^7^ while Parmaksız et al. employed thermoluminescent dosimeters (TLD) and reported doses of 51.80, 47.80, and 9.70 mGy for headache, trauma, and PNS protocols, respectively.^8^ In the present study, eye lens doses across all head CT protocols were higher than those reported by Alwasiah et al.; however, the measurements of the head ATCM protocol showed close agreement with the findings of Parmaksız et al. Conversely, the eye lens doses from all PNS CT protocols were substantially higher than those reported by Parmaksız et al. Several studies revealed that the ODM can achieve eye lens dose reduction ranging from 7.80% to 50.00% compared to the standard head protocols.^16^, ^23^, ^25^, ^35^

Radiation dose is a critical factor affecting CT image quality. Higher‐dose protocols generally improve image quality by reducing quantum noise and enhancing SNR and CNR. In the present study, the fixed tube current technique for head CT examinations yielded the lowest CTDI_vol_ and DLP among the three techniques evaluated. Conversely, for PNS and C‐spine CT examinations, the fixed tube current technique produced the highest CTDI_vol_ and DLP compared to ATCM and ODM techniques. In contrast to previous studies,^26^, ^27^ no statistically significant differences in image noise were observed among the three techniques for head, PNS, and C‐spine CT examinations (*p* > 0.05). The preserved image noise levels are likely attributable to deep learning image reconstruction (DLIR‐M), which effectively suppresses quantum noise relative to conventional reconstruction algorithms.^36^ These findings suggest that advanced reconstruction techniques can successfully compensate for the reduced photon flux in anterior projections during ODM, maintaining diagnostic image quality while achieving substantial eye lens dose reductions.

However, despite comparable noise levels, protocol‐specific differences in CNR were observed. For head CT examinations, ATCM demonstrated statistically significant superior CNR compared to the fixed tube current technique *(p* = 0.029). This finding has important clinical implications, as improved CNR facilitates the detection of subtle pathologies such as early ischemic changes, small hemorrhages, and gray‐white matter differentiation, which are critical diagnostic tasks in emergency neuroimaging. The superior CNR achieved with ATCM, combined with the noise reduction benefits of DLIR, challenges the conventional practice of employing fixed tube current protocols to ensure consistent image quality in emergency trauma patients with potentially suboptimal positioning.

Notably, for head CT examination, the ATCM technique yielded a significantly higher eye lens dose compared to the fixed tube current protocol. This can be explained by both the anatomical positioning of the eye lens and the operational principles of ATCM. The eyeballs are situated within the orbital cavities at the level of the skull base, which is one of the densest anatomical regions in the body. Head CT imaging requires sufficient photon intensity to penetrate these dense cranial structures to achieve adequate image quality for diagnostic evaluation. With the fixed tube current technique, a constant tube current is applied uniformly throughout the entire scanning range. In contrast, the ATCM technique dynamically modulates photon output during acquisition based on patient attenuation to achieve a predetermined target noise index. Meanwhile, the ODM technique selectively reduces tube current to specific anterior regions corresponding to the orbital bone level. As a result, when using the fixed tube current technique, insufficient photon intensity may reach the detector, resulting in increased image noise. Zhao et al. reported that combining ODM with the ASiR‐V reconstruction algorithm more effectively reduces radiation dose in head‐neck CT angiography compared to 3D Smart mA modulation, while maintaining image quality.^37^ Thus, the ODM technique offers the additional advantage of potential reduction of radiation dose to anterior radiosensitive structures, such as the thyroid gland and eye lenses.

Despite the vendor‐specific nature of this study, integrating ODM into routine clinical practice requires careful consideration of scanner capabilities. Although this study utilized a specific vendor's implementation (GE ODM), equivalent organ‐based tube current modulation (OBTCM) technologies are available across major CT platforms (e.g., X‐CARE, Organ Effective Modulation). While absolute dose values may vary by vendor, the fundamental findings regarding the trade‐off between anterior dose reduction and image noise observed in this study are likely applicable to these systems. Medical physicists should therefore integrate these features into routine protocol decisions for PNS and C‐spine imaging. A stepwise implementation is recommended, ensuring that streak artifacts are not induced by the reduced anterior tube current before routine clinical use.

## 5 LIMITATIONS

This study has several limitations. First, the anthropomorphic phantom, PBU‐60, used in this study is a whole‐body CT phantom designed to simulate radiation absorption and Hounsfield unit (HU) values of human tissue for realistic image evaluation. However, the phantom lacks fluid‐ and air‐equivalent structures, particularly in the PNS region. Specifically, the frontal sinuses—normally positioned within the frontal bone superior to the orbits—were absent from the phantom's anatomical representation. This limitation may affect the clinical applicability of our findings,^38^ as tissue heterogeneity influences both dose distribution and objective image quality metrics.

Second, subjective image quality assessment was not performed, limiting the evaluation to objective metrics. Further studies should incorporate observer‐based image quality evaluation to establish the relationship between technical optimization and clinical utility. Multi‐observer studies with experienced radiologists are recommended to evaluate critical factors such as diagnostic confidence, anatomical structure visualization, lesion detectability, image noise perception, and overall diagnostic acceptability.

Third, a notable limitation of this study is the restriction to a 5‐mm slice thickness protocol. While thinner slice acquisitions (e.g., 2 mm) are utilized by some institutions for routine diagnostic imaging, this study prioritized the evaluation of standard protocols currently employed in emergency diagnostic workflows. Crucially, on the CT scanner platform utilized in this study (GE Healthcare), the ATCM and ODM techniques are intrinsically coupled with the primary reconstruction slice thickness and the user‐selected Noise Index (NI). Consequently, reducing the slice thickness without altering the NI would trigger the system to automatically increase the tube current to compensate for the increased quantum noise. This would result in altered radiation doses that do not accurately reflect real‐world clinical practice. Future studies investigating different slice thickness protocols are recommended to further evaluate the dosimetric impact of ODM under varying acquisition parameters.

Finally, this study was conducted using a single CT scanner model without patient movement. While this controlled environment differs from dynamic clinical conditions, the findings regarding scanner applicability remain relevant. Equivalent organ‐based tube current modulation (OBTCM) technologies are currently available across major CT platforms (e.g., X‐CARE, Organ Effective Modulation). Consequently, although absolute dose values may vary by vendor, the fundamental physical findings regarding the trade‐off between anterior dose reduction and image noise are likely applicable to these systems. Therefore, the optimization strategies presented here provide a valid framework for medical physicists integrating these features into routine protocols. Future studies should further investigate ODM performance in patient populations to validate these findings under clinical conditions.

## 6 CONCLUSION

Implementation of the ODM technique for head, PNS, and C‐spine CT examinations effectively reduced radiation dose to the eye lens by 4.28%–47.97%, with no statistically significant differences in image noise, SNR, and CNR (*p* > 0.05) compared to fixed tube current and ATCM techniques. These findings support the feasibility of integrating ODM into routine clinical workflows for head and neck CT examinations in an emergency setting. This technique offers advantages for patient dose optimization by reducing exposure to radiosensitive structures, including the eye lens and other anterior organs, while maintaining diagnostic image quality. Clinical validation studies incorporating subjective image quality assessment are recommended to confirm these preliminary phantom‐based results.

## AUTHOR CONTRIBUTIONS

Wirachad Sripoori, Raksumon Suksi, and Thawatchai Thaikan carried out experiments, data curation and collection. Sowitchaya Huakham prepared figures, tables, and original manuscript. Sowitchaya Huakham and Thanyawee Pengpan contributed through project conceptualization, organization, data analysis, data interpretation, and approved the final version of the manuscript.

## FUNDING

This research received no specific grant from any funding agency in the public, commercial, or not‐for‐profit sectors.

## CONFLICT OF INTEREST STATEMENT

The authors declare no conflicts of interest.

## ETHICS STATEMENT

This study was conducted exclusively on phantoms and did not involve human participants.

## Supporting information



Supporting Information

Supporting Information
