# Supplementary material for: Reduction of radiation dose to the eye lens during common CT examinations of the head, paranasal sinus, and cervical spine in emergency settings: A phantom study
Source: J Appl Clin Med Phys. 2026 Feb 9;27(2):e70486. doi: 10.1002/acm2.70486 (PMC12885872; doi:10.1002/acm2.70486)
Supplement: Supplementary file 1 — Supporting Information [file ACM2-27-e70486-s001.pdf]

Supplementary Table 1 Mean image noise, SNR, and CNR for fixed tube current, ATCM, and ODM techniques.

CT examination	Image noise (HU)			*p*-value	SNR			*p*-value	CNR			*p*-value
	Fixed tube current	ATCM	ODM		Fixed tube current	ATCM	ODM		Fixed tube current	ATCM	ODM	
**Head**	7.02 ± 1.05	6.54 ± 1.05	6.74 ± 1.05	0.098	7.25 ± 0.71	7.97 ± 0.80	7.68 ± 0.77	0.130	4.25 ± 0.39	4.65 ± 0.45	4.37 ± 0.41	0.032
**PNS**	9.14 ± 0.90	9.49 ± 0.89	9.94 ± 0.91	0.294	4.47 ± 0.42	4.39 ± 0.42	4.11 ± 0.39	0.332	2.61 ± 0.24	2.67 ± 0.25	3.25 ± 0.36	0.633
**C-Spine**	7.27 ± 0.92	9.34 ± 1.02	9.69 ± 1.03	0.188*	2.70 ± 0.56	1.97 ± 0.41	1.87 ± 0.37	0.383	1.58 ± 0.27	1.23 ± 0.18	1.21 ± 0.18	0.398*

* Data analyzed using one-way ANOVA; all other parameters were analyzed using the Kruskal-Wallis test.
