# Supplementary material for: Reduction of radiation dose to the eye lens during common CT examinations of the head, paranasal sinus, and cervical spine in emergency settings: A phantom study
Source: J Appl Clin Med Phys. 2026 Feb 9;27(2):e70486. doi: 10.1002/acm2.70486 (PMC12885872; doi:10.1002/acm2.70486)
Supplement: Supplementary file 2 — Supporting Information [file ACM2-27-e70486-s002.docx]

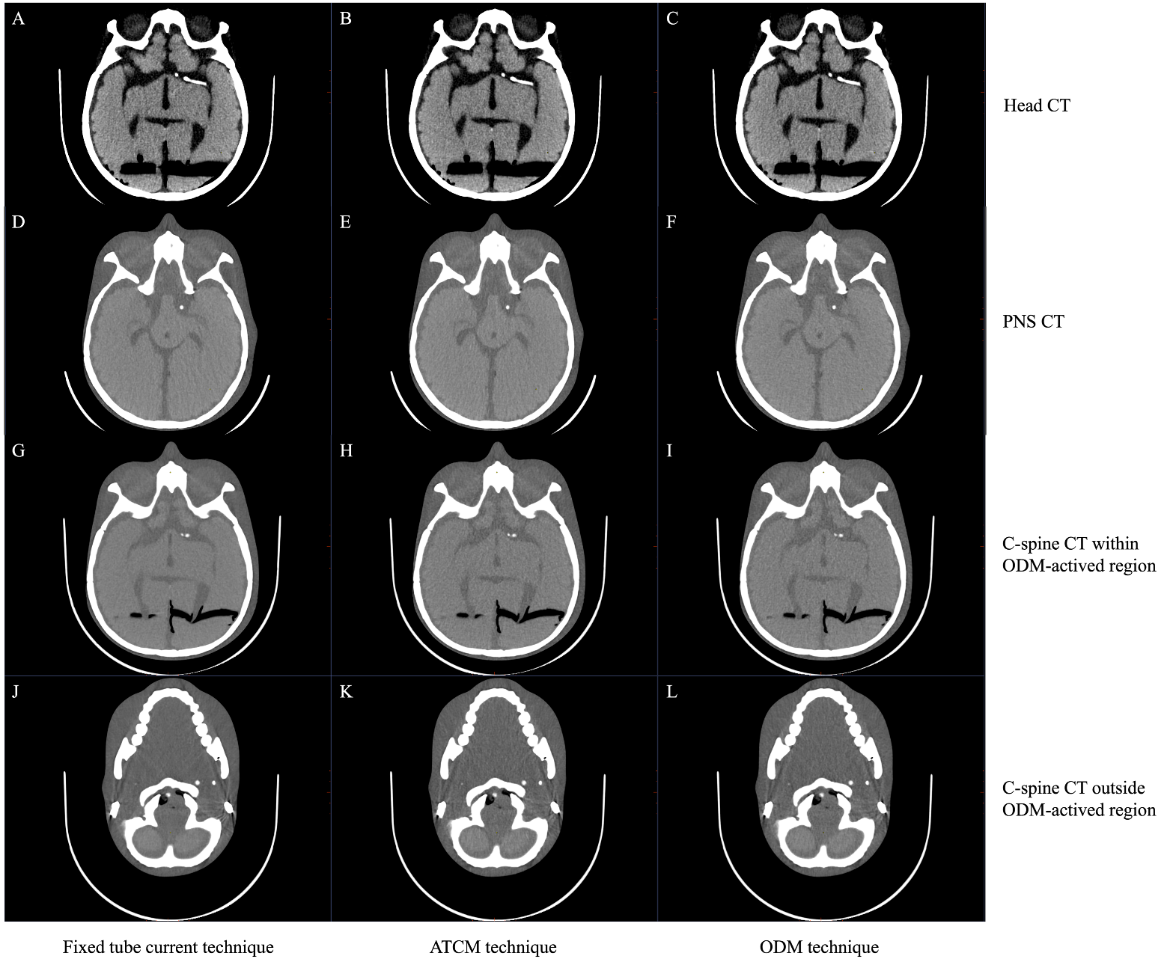
Figure S-1 CT Comparison of axial CT images among fixed tube current, ATCM, and ODM techniques. Upper row (A-C): head CT examination. Middle row (D-F): PNS CT examination. Lower rows (G-L): C-spine CT examination shown at two different levels—within the ODM activated region (G-I) and outside the ODM activated region (J-L). Images are displayed with consistent window settings for direct comparison.
